# Harmonizing the pixel size in retrospective computed tomography radiomics studies

**DOI:** 10.1371/journal.pone.0178524

**Published:** 2017-09-21

**Authors:** Dennis Mackin, Xenia Fave, Lifei Zhang, Jinzhong Yang, A. Kyle Jones, Chaan S. Ng, Laurence Court

**Affiliations:** 1 Department of Radiation Physics, The University of Texas MD Anderson Cancer Center, Houston, TX, United States of America; 2 Graduate School of Biomedical Sciences, The University of Texas Health Science Center at Houston, Houston, TX, United States of America; 3 Department of Imaging Physics, The University of Texas MD Anderson Cancer Center, Houston, TX, United States of America; 4 Department of Diagnostic Radiology, The University of Texas MD Anderson Cancer Center, Houston, TX, United States of America; Institute of Automation Chinese Academy of Sciences, CHINA

## Abstract

Consistent pixel sizes are of fundamental importance for assessing texture features that relate intensity and spatial information in radiomics studies. To correct for the effects of variable pixel sizes, we combined image resampling with Butterworth filtering in the frequency domain and tested the correction on computed tomography (CT) scans of lung cancer patients reconstructed 5 times with pixel sizes varying from 0.59 to 0.98 mm. One hundred fifty radiomics features were calculated for each preprocessing and field-of-view combination. Intra-patient agreement and inter-patient agreement were compared using the overall concordance correlation coefficient (OCCC). To further evaluate the corrections, hierarchical clustering was used to identify patient scans before and after correction. To assess the general applicability of the corrections, they were applied to 17 CT scans of a radiomics phantom. The reduction in the inter-scanner variability relative to non–small cell lung cancer patient scans was quantified. The variation in pixel sizes caused the intra-patient variability to be large (OCCC <95%) relative to the inter-patient variability in 79% of the features. However, with the resampling and filtering corrections, the intra-patient variability was relatively large in only 10% of the features. With the filtering correction, 8 of 8 patients were correctly clustered, in contrast to only 2 of 8 without the correction. In the phantom study, resampling and filtering the images of a rubber particle cartridge substantially reduced variability in 61% of the radiomics features and substantially increased variability in only 6% of the features. Surprisingly, resampling without filtering tended to increase the variability. In conclusion, applying a correction based on resampling and Butterworth low-pass filtering in the frequency domain effectively reduced variability in CT radiomics features caused by variations in pixel size. This correction may also reduce the variability introduced by other CT scan acquisition parameters.

## Introduction

Radiomics studies attempt to stratify patients by variations in quantifiable image features. The impact of these studies is reduced if the variations in image features are caused by differences in the way the images are acquired rather than phenotypical differences in the imaged population. Because many radiomics features relate spatial and intensity information, it would not be surprising to find that these features depend on slice thickness and on the reconstruction field of view (FOV), which determines the image pixel size. Few studies have directly investigated the impact of slice thickness and pixel size on radiomics features [1–5]. This is surprising, as both image thickness and pixel size are routinely adapted on a patient-by-patient basis in diagnostic imaging to optimize radiation dose and image quality. For example, in a study of 74 patients with lung cancer, Basu et al. found that the reconstructed slice thickness in computed tomography (CT) varied from 3 to 6 mm and that the pixel size varied from 0.59 to 0.94 mm [6, 7]. In another study of CT texture features in 39 patients with metastatic renal cell cancer, the pixel sizes ranged from 0.59 to 0.78 mm [8]. In a third study in which CT radiomics features were used to classify interstitial pneumonias, the pixel size ranged from 0.39 to 0.82 mm [9], and the authors resampled the images to produce isotropic 0.59-mm^3^ voxels to ensure consistency of the physical dimensions. Unfortunately, many radiomics studies do not report the pixel size or reconstruction FOV, even when reporting other scan acquisition and reconstruction parameters.

To date, most radiomics studies have focused on clinical applications. However, researchers have begun to analyze the robustness of radiomics features. Test/retest studies have measured the reproducibility of features calculated on the same patient using the same scanner [10–13]. Fave et al. investigated the reproducibility of features in cone beam CT and found that some features are robust, but only if the imaging protocol is consistent and the motion is limited to less than 1 cm. In another study, Fave et al. found that 12 of 28 features changed significantly when calculated using average images rather than end-of-exhale phase images in breath-hold CT; they also found that a simulated decrease in tube voltage had a negligible impact on texture features while a simulated decrease in tube current significantly changed 13 of 23 texture features [14]. In a phantom-based study, Mackin et al. showed that varying the scan acquisition parameters produced significant variation in the calculated features; however, because the scan parameters were inconsistent, the parameters that caused the variation remained unknown [15]. Zhao et al. investigated the effects of smooth and sharp CT reconstruction algorithms for several image thicknesses and determined that the algorithms should not be used interchangeably [1]. Other studies have investigated the robustness of texture features in FDG PET images [16] and the robustness of image segmentation methods [17]. These studies identify problems but do not specify the solutions.

Implementing a scanning protocol to enforce consistency in the acquisition parameters might provide a solution for prospective studies. Alternatively, scans could be reconstructed more than once, and the additional reconstructions could use standardized values for the slice thickness and FOV. However, the majority of radiomics studies are retrospective and limited to images that were not acquired according to a protocol. Methods are needed to standardize, or *harmonize*, images to enable to comparison of images taken using a variety of scanner models and acquisition parameters.

The image pixel size is of particular importance to texture analysis features, which relate the spatial and intensity information in images. Consider for example a 5×5 pixel neighborhood, used for neighborhood gray-tone difference matrix features [18]. In a 512×512 pixel image reconstructed with a 50-cm FOV, a 5×5 pixel area represents a physical area of 24 mm; in a 512×512 pixel image reconstructed with a 30-cm FOV, however, a 5×5 pixel neighborhood represents a physical area of only 14 mm, an area decrease of 42%.

The purpose of our study was to develop a correction technique to reduce or eliminate the variability in radiomics features due to differences in image pixel size. Our efforts focus on image resampling and on Butterworth filtering in the frequency domain. We evaluated this correction method using patient CT scans that have been reconstructed using multiple pixel sizes. We applied the same method to CT scans of a Credence Cartridge Radiomics (CCR) radiomics phantom using a variety of scanners and scan parameters.

## Materials and methods

### FOV effects data set

All clinical investigation were conducted according to the principles expressed in the Declaration of Helsinki. The data used in this study was obtained with approval of the University of Texas MD Anderson Cancer Center's Institutional Review Board. The data was initially obtained with written, informed consent. The IRB waved written, informed consent for the retrospective study of this data. We collected raw, unreconstructed data from the CT scans of 8 lung cancer patients performed between July 2004 and October 2005. The scans were performed using a GE Medical Systems LightSpeed 16 CT scanner (GE Healthcare, Milwaukee, WI). All scans were performed in accordance with the Declaration of Helsinki. The scans were acquired with 396 mAs (6 scans) or 167 mAs (2 scans) and 120 kVp (all scans) without contrast. The slice thickness was 2.5 mm. For each patient, we reconstructed the scans with the same set of reconstruction parameters except that we varied the reconstruction FOVs from 30 to 50 cm in increments of 5 cm to produces pixel sizes of 0.59 to 0.98 mm. Thus, this data set consisted of 8 CT scans with 5 reconstructions for a total of 40 DICOM series. The size of this retrospective study was limited by the number of preserved, unreconstructed CT scan data files. The patients comprised 2 women and 6 men. Their mean age was 53 years (range, 21–65 years), their mean height was 175 cm (range, 163–183 cm), their mean weight was 84.6 kg (range, 68.0–98.2 kg), and their mean body mass index was 27.7 (range, 20.8–35.5).

### Radiomics features

We examined the effects of the filtering on 5 categories of features: image intensity, shape, gray level co-occurrence matrix (GLCM), gray level run length (GLRL), and neighborhood gray-tone difference matrix (NGTDM). Image intensity features are calculated directly from the images and include conventional metrics such as the mean, median, and standard deviation. Shape features describe the basic geometry of the region of interest (ROI). GLCM features are derived from the spatial and angular relationships of the differences in the image intensity [19]. GLRL features describe patterns in the “run length,” or number of consecutive voxels with the same intensity [20]. NGTDM features describe human-perceptible features such as coarseness and contrast [18]. To calculate the features, we used IBEX radiomics software [21]. Rather than calculating the features for the full, 3-dimensional images, we calculated the features for each 2-dimensional slice and then combined the results, a method referred to in IBEX as 2.5D calculation.

### Image preprocessing methods

Decreasing the image reconstruction FOV increases the image resolution and the noise per pixel, both of which can affect the feature values. GLCM, GLRL, and NGTDM features inherently depend on the spatial resolution, and all features can be affected by image noise. The CT images used in this study had 512×512 pixels per slice. The resolution is inversely proportional to pixel size and to the reconstruction FOV ($\mathrm{pixel}\;\mathrm{size}=\frac{\mathrm{FOV}}{\mathrm{number}\;\mathrm{of}\;\mathrm{pixels}}$). Images created with smaller FOVs have a higher resolution and thus contain more high-frequency information and noise. Therefore, we first resampled the images using bilinear interpolation to a uniform 1 mm/pixel for all images. To reduce the information discrepancy, we filtered each slice of the ROI in frequency space using 2D, second-order Butterworth low-pass filters [22, 23]. The ROI images for each CT slice were padded to 512×512 pixels before the filters were applied. We applied the filters after resampling to ensure that the frequency content was weighted consistently in each image set. In total, 7 different correction levels were tested (Table 1).

**Table 1:** Image correction level.

Correction level	Resampling	Filter (order, frequency cutoff)
1	None	None
2	1 mm/pixel	None
3	None	BW(2, 100)
4	1 mm/pixel	BW(2, 200)
5	1 mm/pixel	BW(2, 125)
6	1 mm/pixel	BW(2, 100)
7	1 mm/pixel	BW(2, 75)

The 7 resampling and filtering combinations used as corrections in the study. BW indicates Butterworth.

### Quantifying the inter- and intra-patient variability

Variations in the CT pixel size can affect the calculated feature values of patient images. To quantify this effect, we used the overall concordance correlation coefficient (OCCC), introduced by Barnhart et al. [24]. The OCCC assesses the agreement of a single measured value (in this case, radiomics features) with multiple subjects (patients) by multiple observers (reconstruction FOVs). The OCCC, *ζ*, is given by

$$\zeta =\frac{2\sum_{j=1}^{J-1}\sum_{k=j+1}^{J}S_{jk}}{(J-1)\sum_{J=1}^{J}S_{j}^{2}+J\sum_{j=1}^{J}({\bar{Y}}_{j}-\bar{Y}{)}^{2}}$$ (1)

where *J* is number of FOVs, *S*_*jk*_ is the covariance of FOVs *j* and *k*, *S*_*j*_ is the sample standard deviation for FOV *j*, ${\bar{Y}}_{j}$ is the mean value of FOV *j*, and $\bar{Y}$ is the mean value of the means for each FOV. We calculated the OCCC using the R software package [25].

To further evaluate the correction techniques, we used hierarchical clustering to form 8 groups of the 40 CT image samples (8 patients × 5 pixel sizes), each group representing 1 patient. We used the hierarchical clustering software from R’s stats package with the Euclidean distance metric. Eight clusters that each comprise 5 CT images for 1 patient would indicate pixel size effects that are small relative to the inter-patient variability; clusters comprising CT images for different patients would indicate pixel size effects that are large relative to the inter-patient variability. In this study, we considered 3 features that have been shown to be prognostic in CT radiomics studies: entropy, busyness, and gray level non-uniformity. Entropy has been found to be predictive of tumor recurrence after stereotactic ablative radiotherapy [26] and to identify changes in apparently disease-free areas of the liver in patients with hepatic metastases [27]. Busyness has been found to be predictive of distant metastases in patients with stage III non–small cell lung cancer (NSCLC) [28]. Gray level non-uniformity has been shown to separate NSCLC patients into survival groups in a statistically significant manner [12]. These 3 features also represent 3 feature categories: intensity histogram, NGTDM, and GLRL matrix. The clustering was performed on images with and without pixel size corrections.

### Correcting for inter-scanner variability

**Fig 1:**
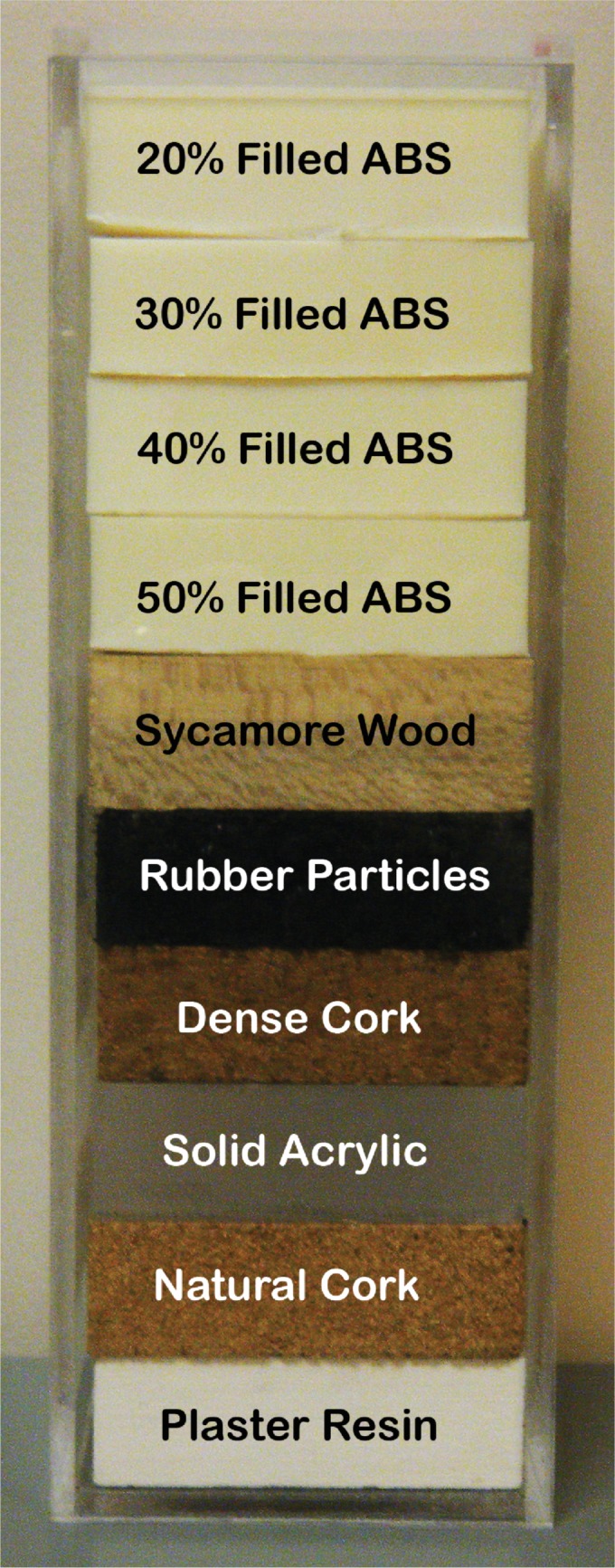
The credence cartridge radiomics phantom.

We applied the same pixel size corrections to the CCR phantom (Fig 1) to determine whether they are generally applicable to the problem of mitigating the effects of inter-scanner variability. In a previous study, the CCR phantom was scanned 17 times on 16 different scanners from GE Healthcare (Milwaukee, WI, USA), Philips Healthcare (Amsterdam, Netherlands), Siemens Healthineers (Erlangen, Germany), and Toshiba Medical Systems (Otawara, Japan) [15]. The CCR phantom comprises 10 cartridges of natural and man-made materials, each with a different texture. In the present study, we used the rubber particle, dense cork, and sycamore wood cartridges. Of these, the rubber particle cartridge is the most similar to NSCLC tumors in CT number and standard deviation. The dense cork cartridge has textures similar to those of human tissues. The sycamore wood cartridge has natural run-length textures. For each cartridge, we calculated the 150 radiomics features and applied the same 7 preprocessing methods on 16 cubic ROIs, each with a volume of 8 cm^3^. For each scan, we reported the mean feature values in each cartridge to reduce the effects of small setup errors when the phantom is scanned. The scans were acquired using the chest protocols commonly used with each individual scanner in routine clinical practice. The differences in these protocols produced a range of acquisition parameters for the reconstruction FOVs (25–52 cm; pixel sizes 0.49–1.01 mm), the slice thicknesses (2.0–3.0 mm), and the effective mA∙s (17–1102). All the scans used 120 kVp.

The inter-scanner variability in radiomics features is not concerning unless it is large enough to be similar to the variability in patients. We therefore compared the inter-scanner variability of the radiomics features of the phantom scans with the variability found in a sample of 20 NSCLC patients (10 women and 10 men). The mean age of the patients was 67 years (range, 53–78 years). The patients’ mean body mass index was 25.3 (range, 13.1–33.3); their heights ranged from 154 to 182 cm, and their weights ranged from 41.0 to 97.6 kg. One patient’s data were not included in the means because the patient requested that access to those clinical data be restricted. These scans were acquired using a GE Healthcare Discovery ST scanner with a slice thickness of 2.5 mm and a pixel size of 0.98 mm. The scans were acquired using 50 mA∙s and 120 kVp. All scans were performed in accordance with the Declaration of Helsinki.

To quantify the effect of the corrections for an individual scanner *j*, we calculated the scaled features, $\hat{f_{ij}}$, as

$$\hat{f_{ij}}=\frac{f_{ij}-\langle f_{i}\rangle }{\sigma_{fi}(NSCLC)}$$ (2)

where *f*_*ij*_ is the feature value for preprocessing method *i* and scanner *j*, ⟨*f*_*i*_⟩ is the average feature value for preprocessing method *i*, and *σ*_*fi*_(*NSCLC*) is the standard deviation of the feature *f* in the patient population after preprocessing of the images with method *i*.

To measure the effects of the corrections on the inter-scanner variability, we calculated the scaled variability, ${\hat{v}}_{i}$, given by

$${\hat{v}}_{i}=\frac{{\sigma_{f}}_{i}}{\sigma_{fi}(NSCLC)}$$ (3)

where ${\sigma_{f}}_{i}$ is the standard deviation of feature *f* after treatment *i* for the phantom scans and *σ*_*fi*_(*NSCLC*) is again the standard deviation of the feature *f* in the patient population after correction of the images with method *i*. Using the standard deviation of the features in the patients penalizes corrections that reduce the variability in the patient sample more than the variability in the phantom scans.

## Results

### Effects of FOV and preprocessing

The OCCC values for 150 features calculated for the patient FOV combinations are summarized in Table 2. Because we compared features calculated using the same scans, OCCC values close to 1 would be reasonable. Of the 7 correction levels we tested, the lowest mean OCC values for all features were produced by no preprocessing (0.87) and by resampling without filtering (0.84). The OCCC values for features derived from the shape of the ROI before preprocessing were all very close to 1. Therefore, the corrections had little effect on these feature values. This result for shape features was expected because the ROIs do not depend on preprocessing. Resampling plus filtering improved the average OCCC values for the remaining 4 feature categories. Resampling and filtering with a Butterworth order 2, cutoff 125 filter, abbreviated BW(2, 125), increased the fraction of features with OCCC values of >0.95 from 0.21 to 0.99. A BW(2, 75) filter produced the largest fraction of features with OCCC values of >0.99 (0.75). In general, resampling plus low-pass filtering either improved or did not affect the OCCC values. Resampling or low-pass filtering alone produced mixed results, sometimes making the OCCC values worse and sometimes making them slightly better. The OCCC results for images processed using Gaussian and mean filters instead of Butterworth filters are provided in S1 Table.

**Table 2:** Summary of the OCCC values for 150 radiomics features.

	Intensity Histogram	GL Co-occurrence	GL Run Length	NGTDM	Shape	All Features
	No. of Features					
	11	110	12	5	12	150
Pixel Size Correction	Mean OCCC Value					
1) None	0.73	0.89	0.83	0.91	1.00	0.87
2) 1 mm/pixel	0.96	0.83	0.76	0.96	1.00	0.84
3) BW(2, 125)	0.76	0.94	0.86	0.88	1.00	0.91
4) 1 mm/pixel; BW(2, 200)	0.98	0.95	0.94	0.98	1.00	0.95
5) 1 mm/pixel; BW(2, 125)	0.98	0.99	0.99	0.99	1.00	0.99
6) 1 mm/pixel; BW(2, 100)	0.95	0.99	1.00	0.99	1.00	0.99
7) 1 mm/pixel; BW(2, 75)	0.98	0.99	1.00	0.99	1.00	0.99
Pixel Size Correction	Fraction of OCCC Values > 0.95					
1) None	0.18	0.23	0.00	0.20	1.00	0.21
2) 1 mm/pixel	0.73	0.36	0.17	1.00	1.00	0.41
3) BW(2, 125)	0.27	0.36	0.33	0.40	1.00	0.36
4) 1 mm/pixel; BW(2, 200)	1.00	0.70	0.58	1.00	1.00	0.73
5) 1 mm/pixel; BW(2, 125)	0.91	1.00	1.00	1.00	1.00	0.99
6) 1 mm/pixel; BW(2, 100)	0.45	0.95	1.00	1.00	1.00	0.91
7) 1 mm/pixel; BW(2, 75)	1.00	0.87	1.00	1.00	1.00	0.90
Pixel Size Correction	Fraction of OCCC Values > 0.99					
1) None	0.09	0.14	0.00	0.00	1.00	0.13
2) 1 mm/pixel	0.27	0.17	0.00	0.00	1.00	0.17
3) BW(2, 125)	0.00	0.00	0.00	0.00	0.75	0.01
4) 1 mm/pixel; BW(2, 200)	0.36	0.18	0.00	0.20	1.00	0.19
5) 1 mm/pixel; BW(2, 125)	0.45	0.58	0.67	0.60	0.92	0.59
6) 1 mm/pixel; BW(2, 100)	0.09	0.69	1.00	0.60	0.92	0.67
7) 1 mm/pixel; BW(2, 75)	0.45	0.75	1.00	0.60	0.92	0.75

Where indicated, images were resampled to 1 mm/pixel and were filtered with a Butterworth filter; the values in parenthesis indicate order and the cutoff frequency. GL indicates gray level; NGTDM, neighborhood gray-tone difference matrix; BW, Butterworth; OCCC, overall concordance correlation coefficient.

### Effect of pixel size on hierarchical clustering

The hierarchical clusters from the sets of 40 CT images (8 patients × 5 FOVs each) are shown in Fig 1. The clusters were calculated using the features entropy, busyness, and gray level non-uniformity. Without the pixel size correction, the 5 FOV scans were correctly grouped together for only 2 patients. After resampling of the images to 1 mm/pixel, the 5 FOV scans were correctly grouped together for 4 patients. However, with correction level 5—i.e., resampling and filtering with a BW(2, 125) filter—the 5 FOV scans were correctly grouped for all 8 patients, indicating that the correction effectively mitigated the effects of the variable pixel sizes.

### Effects of preprocessing on inter-scanner variability

**Fig 2:**
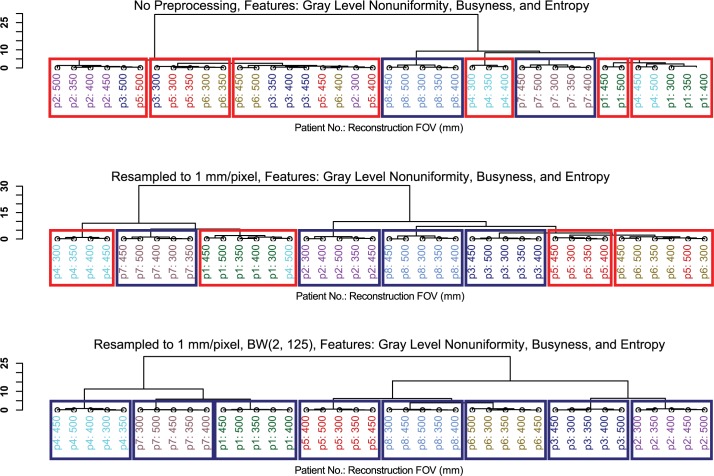
Hierarchical clusters of lung cancer patient CT scans using the Euclidean distance of the features entropy, busyness, and gray level non-uniformity. The features were extracted from images that had (a) no preprocessing, (b) resampling to 1 mm/pixel, and (c) resampling to 1 mm/pixel and filtering with a Butterworth filter (order 2, frequency cutoff 125). Boxes indicate incorrect (red) and correct (blue) groupings of the 5 FOV scans for each patient.

We also calculated the same 150 features and applied the same corrections to 17 scans of the CCR radiomics phantom. The effects of the corrections on the scaled-feature (Eq 2) contrast for each of the phantom scans are shown in Fig 2. For uncorrected images (Fig 2A), the average scaled variability was 0.51. Resampling the images to 1 mm/pixel without filtering (Fig 2B) increased the mean scaled variability of contrast to 0.96. Applying correction level 7—i.e., resampling to 1 mm/pixel, and applying a BW(2, 75) filter—reduced the mean scaled variability for the 17 scanners to 0.20 (Fig 2C). Thus, with the correction, the variability between the scanners was small relative to the variability between the patients. Resampling and filtering do have a strong effect on the variability of feature values extracted from the patient CT scans. The standard deviation for contrast, for example, was 1.6 without preprocessing, 1.4 after resampling to 1 mm/pixel, and 0.5 after resampling applying a BW(2, 75) filter. This indicates that there may be information loss with preprocessing and that standardizing the CT scan acquisition protocols before image reconstruction may be a better approach to image harmonization than resampling and filtering afterwards.

**Fig 3:**
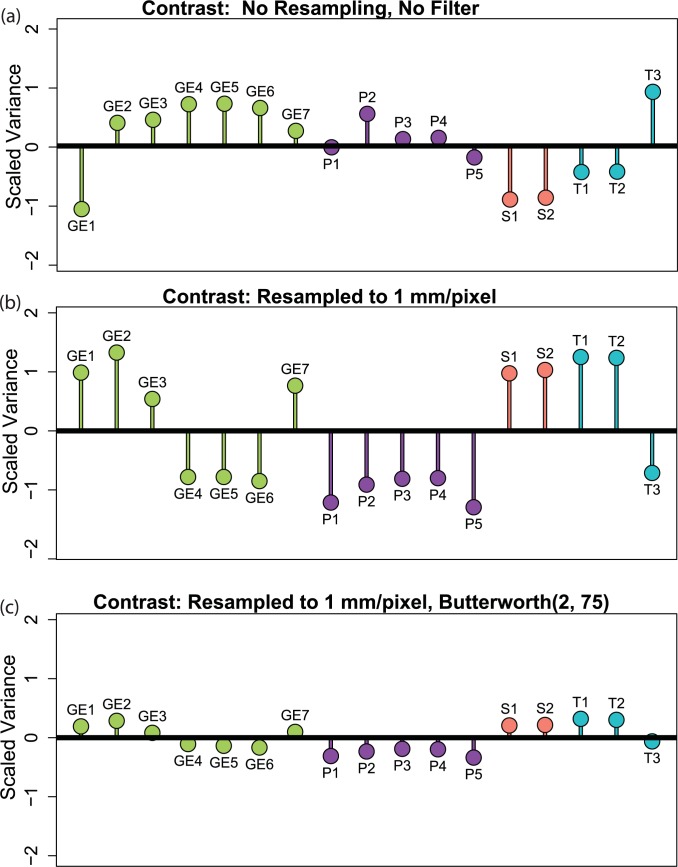
Scaled contrast. Scaled contrast for the CCR phantom’s rubber particle cartridge scanned with 17 different CT scanner configurations. (a) Feature values without image preprocessing. (b) Feature values calculated after all images had been resampled to 1 mm/pixel. (c) Feature values calculated after all images had been resampled to 1 mm/pixel and filtered with Butterworth filter (order 2, frequency cutoff 75). The points are color coded and labeled according to the manufacturer of the scanner: GE indicates GE Healthcare (green); P, Philips Healthcare (purple); S, Siemens Healthineers (pink); T, Toshiba Medical Systems (cyan).

**Fig 4:**
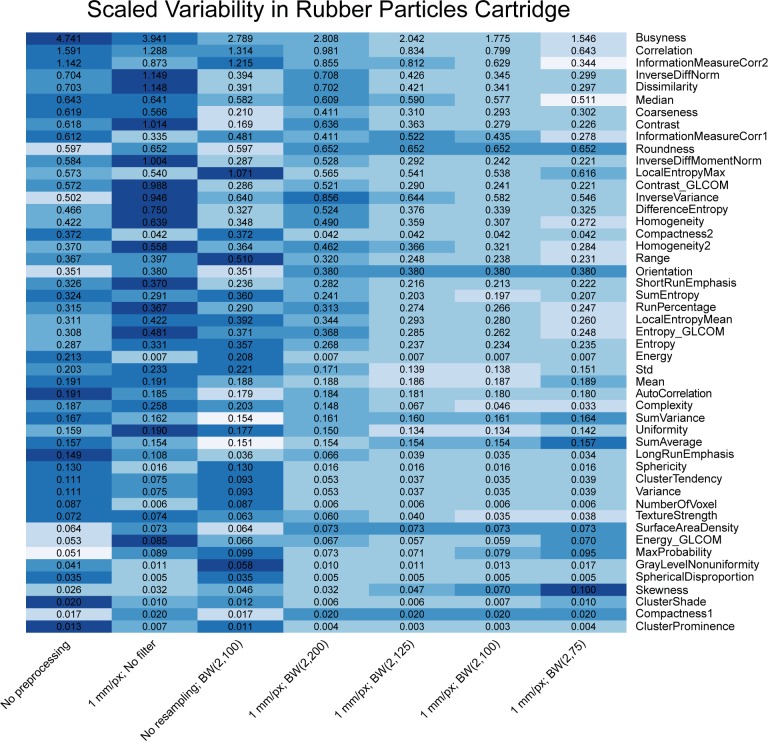
Heat map comparing the scaled variability for the 7 pixel size correction levels for the rubber particle cartridge of the CCR phantom. The features were calculated for the phantom for 17 scans. The color is rescaled on a row-by-row basis; darker colors indicate more variability. The values in the cells are the scaled variability values. BW indicates Butterworth; px, pixel.

**Fig 5:**
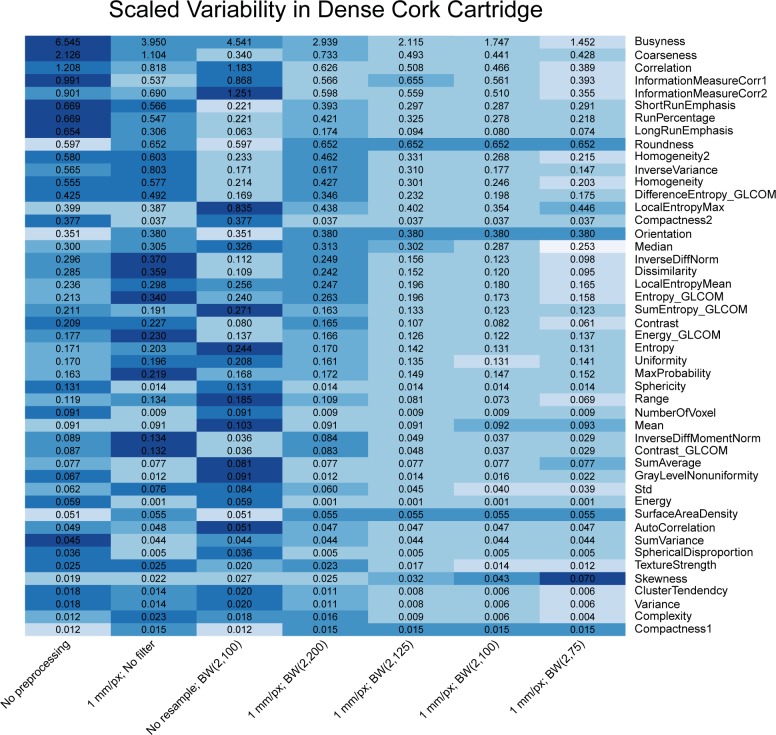
Heat map comparing the scaled variability for the 7 pixel size correction levels for the dense cork cartridge of the CCR phantom. The features were calculated for the phantom for 17 scans. The color is rescaled on a row-by-row basis; darker colors indicate more variability. The values in the cells are the scaled variability values. BW indicates Butterworth; px, pixel.

**Fig 6:**
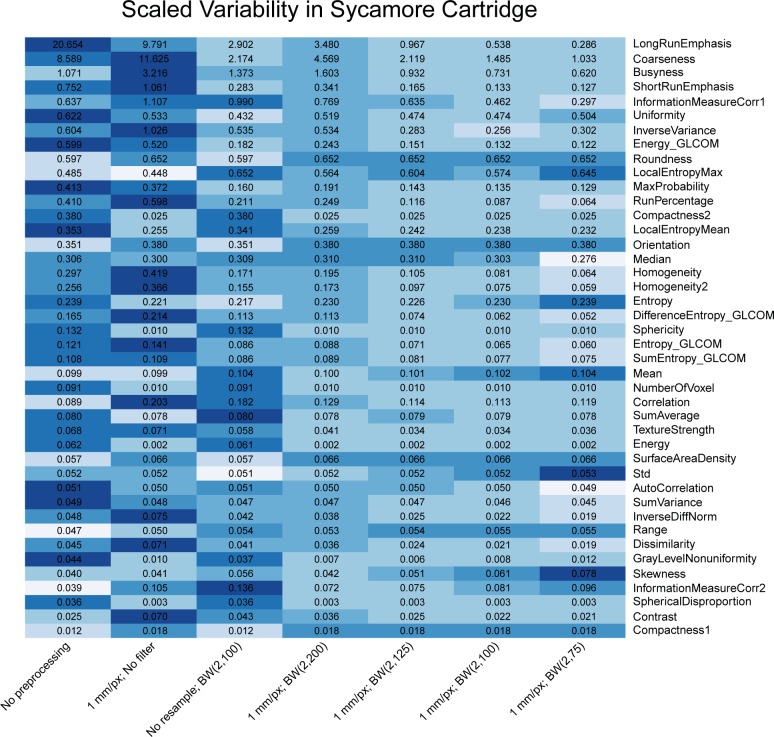
Heat map comparing the scaled variability for the 7 pixel size correction levels for the sycamore wood cartridge of the CCR phantom. The features were calculated for the phantom for 17 scans. The color is rescaled on a row-by-row basis, and darker colors indicate more variability. The values in the cell are the scaled variability values. BW indicates Butterworth; px, pixel.

Heat maps comparing the scaled variability (Eq 3) of features’ correction levels are shown in Figs 3–5. The features were calculated for CT scans of the rubber particle (Fig 3), dense cork (Fig 4), and sycamore (Fig 5) cartridges. GLCM and GLRL are calculated for directions 0°, 45°, 90°, and 135°. The results for each direction are also summed. Only these summary features are included in the heat map. Features with scaled variability of <0.01 were not included in the results. In general, the scaled variability decreased with increasing correction level. For example, for the rubber particle cartridge, resampling and filtering the images with correction level 7—resampling with a BW(2, 75) filter—reduced the variability by at least 20% for 61% of the features and increased the variability by at least 20% for only 6% of the features. On the other hand, correction level 2—resampling without filtering—decreased the scaled variability by at least 20% for 27% of the features and increased the variability 20% for 31% of the features. Thus, in more cases than not, resampling alone produced more variability than no preprocessing at all. We found that resampling alone often increased the variability in the dense cork and sycamore cartridges as well. The changes in the scaled variability upon resampling without filtering were decreases in 30% and increases in 26% of the features in the dense cork and decreases in 19% and increases in 36% of the features in the sycamore. The Butterworth filter with cutoff frequency of 200 produced a relatively small effect (Figs 4–6). However, there was little difference between the effects of cutoff frequencies, 75, 100, and 125. Not surprisingly, the features with the largest scaled variability were more effected by preprocessing. For example, the scaled variability of busyness, extracted from the rubber particles cartridges, was reduced from 4.7 to 1.5 (68%), and correlation was reduced from 1.6 to 0.6 (62%). In the dense cork, busyness was reduced from 6.5 to 1.5 (77%), and in the sycamore wood, longRunEmphasis was reduced from 20.7 to 0.286 (99%).

## Discussion

We found that resampling and low-pass filtering of CT images could correct much of the variability in quantitative features due to inconsistent image pixel sizes. Preprocessing the images increased the OCCC to values near 1, indicating that the effects of the varied FOV had been greatly reduced. Hierarchical clustering supported this result. Only after the corrections that included both resampling and filtering were the 5 FOV scans for each patient correctly grouped together. For scans of a phantom on multiple scanners with a variety of acquisition parameters, the variability of the features calculated for the rubber particle, dense cork, and sycamore wood cartridges was also generally reduced by the corrections. The only cost of this reduction in variability was the computing time needed to process the images.

A seemingly logical preprocessing step is to resample all images in a study so that they all have the same resolution, and this approach has been used in previous studies [9, 15]. Surprisingly, we found in our patient study that resampling alone produces more variability (smaller average OCCC values) for both GLCM and GLRL features compared with no preprocessing. Also, for most radiomics features in the phantom study, the scaled variability values produced with resampling were larger than those produced with no preprocessing. A possible explanation for this increased variability is that resampling to a smaller number of pixels/mm itself acts as a low-pass filter whose strength depends on the initial resolution. Therefore, images with different pixel sizes have different filter strengths, producing differences in the radiomics features. Another possible explanation for the increased variability is aliasing, in which high frequencies produce spurious low-frequency responses in down-sampled images.

In our patient study in which the FOV was controlled, we found that the corrections could reduce the effects of the variable pixel sizes. A limitation of this study was the number of patients. Only 8 unreconstructed CT scans were available. A larger patient cohort might have enabled the study to determine which of the filtering levels is the most effective or even an optimal filtering level. In our phantom study, the corrections reduced the scaled variability, but we were not able to isolate the source of the variability. Future studies in which the scan parameters are more tightly controlled would enable the identification of the sources of the variability.

To the best of our knowledge, no other studies have investigated resampling and low-pass filtering specifically as a way to correct for pixel size effects. However, some previous studies have investigated applying low-pass and band-pass filters to images before calculating the radiomics features. Miles et al. used Gaussian image filters to highlight image features according to size [29]. Ganeshan et al. used Gaussian filters combined with a Laplacian edge detector (LoG) to highlight features in a dynamic contrast-enhanced CT study of colorectal cancer [30]. Other studies also found that LoG filtering increased the significance of some features [8, 31]. In the present study, we did not compare the Butterworth filter to other low-pass filters. Also, we did not consider the effects of other image-enhancement techniques, such as edge enhancement, as the performance of such techniques is likely affected by resampling and filtering. Future studies may focus on how resampling and low-pass filtering can be optimized for use in conjunction with image-enhancement techniques.

Future studies should also focus on identifying the optimal application of resampling and filtering to a patient sample that contains images produced with a range of pixel sizes. Our results suggest that resampling and filtering increased improve the significance of radiomics features when used in statistical modeling, but this expected result has not been demonstrated. Similarly, our study was not designed to identify the best preprocessing method. The best preprocessing method likely will be application dependent, and some experimentation with preprocessing parameters will become an essential step in radiomics model building.

The National Cancer Institute has expressed the need for standards in quantitative imaging [32]. The results of the present study provide a starting point for establishing standards of image harmonization, a necessary step when comparing images taken using a variety of CT scanning protocols. Standardizing image preprocessing is a necessary step towards standardizing the image features themselves.

## Conclusions

In conclusion, applying a correction based on resampling and Butterworth low-pass filtering in the frequency domain effectively reduces the variability in CT radiomics features caused by variations in the pixel size. This correction may also reduce the variability introduced by other CT scan acquisition parameters.

## Supporting information

S1 Table Summary of the OCCC values for 138 radiomics features. This table supports Table 2 in the primary text and shows the results for Gaussian and mean low pass filters rather than Butterworth filters. As indicated in the first column, images were resampled to 1 mm/pixel and were filtered with a mean or Gaussian filter. The masks used to apply the filters to the image pixels were either 3x3 pixels or 5x5 pixels as indicated. The Gaussian filter widths were either 1 or 3 pixels as indicated by the sigma values. GL indicates gray level; NGTDM, neighborhood gray-tone difference matrix; BW, Butterworth; OCCC, overall concordance correlation coefficient.
(DOCX)

S1 Fig Hierarchical clusters of lung cancer patient CT scans using the Euclidean distance of the features entropy, busyness, and gray level non-uniformity. The features were extracted from images that had (a) no resampling with a Butterworth filter (order 2, frequency cutoff 100), (b) resampling to 1 mm/pixel and filtering with a Butterworth filter (order 2, frequency cutoff 200), (c) resampling to 1 mm/pixel and filtering with a Butterworth filter (order 2, frequency cutoff 125), (d) resampling to 1 mm/pixel and filtering with a Butterworth filter (order 2, frequency cutoff 100), and (e) resampling to 1 mm/pixel and filtering with a Butterworth filter (order 2, frequency cutoff 75). Boxes indicate incorrect (red) and correct (blue) groupings of the 5 FOV scans for each patient.
(EPS)

S2 Fig Hierarchical clusters of lung cancer patient CT scans using the Euclidean distance of the features entropy, busyness, and gray level non-uniformity. The features were extracted from images that had (a) no preprocessing, (b) resampling to 1 mm/pixel, (c) resampling to 1 mm/pixel and filtering with a 3x3 pixel mean filter, (d) resampling to 1 mm/pixel and filtering with a 3x3 pixel, 1 mm width Gaussian filter, and (e) resampling to 1 mm/pixel and filtering with a 5x5 pixel, 3 mm width Gaussian filter. Boxes indicate incorrect (red) and correct (blue) groupings of the 5 FOV scans for each patient.
(EPS)
