# Correction: Harmonizing the pixel size in retrospective computed tomography radiomics studies

**DOI:** 10.1371/journal.pone.0191597

**Published:** 2018-01-17

**Authors:** Dennis Mackin, Xenia Fave, Lifei Zhang, Jinzhong Yang, A. Kyle Jones, Chaan S. Ng, Laurence Court

S1 Table, S1 and S2 Figs appear incorrectly. The corrected files can be viewed below.

## Supporting information

S1 Table Summary of the OCCC values for 138 radiomics features. This table supports Table 2 in the primary text and shows the results for Gaussian and mean low pass filters rather than Butterworth filters. As indicated in the first column, images were resampled to 1 mm/pixel and were filtered with a mean or Gaussian filter. The masks used to apply the filters to the image pixels were either 3x3 pixels or 5x5 pixels as indicated. The Gaussian filter widths were either 1 or 3 pixels as indicated by the sigma values. GL indicates gray level; NGTDM, neighborhood gray-tone difference matrix; BW, Butterworth; OCCC, overall concordance correlation coefficient.
(PDF)

S1 Fig Hierarchical clusters of lung cancer patient CT scans using the Euclidean distance of the features entropy, busyness, and gray level non-uniformity. The features were extracted from images that had (a) no resampling with a Butterworth filter (order 2, frequency cutoff 100), (b) resampling to 1 mm/pixel and filtering with a Butterworth filter (order 2, frequency cutoff 200), (c) resampling to 1 mm/pixel and filtering with a Butterworth filter (order 2, frequency cutoff 125), (d) resampling to 1 mm/pixel and filtering with a Butterworth filter (order 2, frequency cutoff 100), and (e) resampling to 1 mm/pixel and filtering with a Butterworth filter (order 2, frequency cutoff 75). Boxes indicate incorrect (red) and correct (blue) groupings of the 5 FOV scans for each patient.
(PDF)

S2 Fig Hierarchical clusters of lung cancer patient CT scans using the Euclidean distance of the features entropy, busyness, and gray level non-uniformity. The features were extracted from images that had (a) no preprocessing, (b) resampling to 1 mm/pixel, (c) resampling to 1 mm/pixel and filtering with a 3x3 pixel mean filter, (d) resampling to 1 mm/pixel and filtering with a 3x3 pixel, 1 mm width Gaussian filter, and (e) resampling to 1 mm/pixel and filtering with a 5x5 pixel, 3 mm width Gaussian filter. Boxes indicate incorrect (red) and correct (blue) groupings of the 5 FOV scans for each patient.
(PDF)
