# Supplementary material for: Harmonizing the pixel size in retrospective computed tomography radiomics studies
Source: PLoS One. 2017 Sep 21;12(9):e0178524. doi: 10.1371/journal.pone.0178524 (PMC5608195; doi:10.1371/journal.pone.0178524)
Supplement: S1 Table — This table supports Table 2 in the primary text and shows the results for Gaussian and mean low pass filters rather than Butterworth filters. As indicated in the first column, images were resampled to 1 mm/pixel and were filtered with a mean or Gaussian filter. The masks used to apply the filters to the image pixels were either 3x3 pixels or 5x5 pixels as indicated. The Gaussian filter widths were either 1 or 3 pixels as indicated by the sigma values. GL indicates gray level; NGTDM, neighborhood gray-tone difference matrix; BW, Butterworth; OCCC, overall concordance correlation coefficient. (DOCX) [file pone.0178524.s001.docx]

|  | Intensity Histogram | GL Co-occurrence | GL Run Length | NGTDM | All Features |
| --- | --- | --- | --- | --- | --- |
|  |  |  |  |  |  |
|  | No. of Features | | | | |
|  | 11 | 110 | 12 | 5 | 138 |
|  |  |  |  |  |  |
| Pixel Size Correction | Mean OCCC Value | | | | |
| 1) None | 0.73 | 0.89 | 0.83 | 0.91 | 0.87 |
| 2) 1 mm/pixel | 0.95 | 0.83 | 0.76 | 0.96 | 0.84 |
| 3) Gaussian(sigma=3, mask=5x5) | 0.75 | 0.94 | 0.88 | 0.85 | 0.92 |
| 4) 1 mm/pixel; mean(3x3) | 0.98 | 0.98 | 0.99 | 0.98 | 0.98 |
| 5) 1 mm/pixel; Gaussian(1, 3x3) | 0.99 | 0.98 | 0.99 | 0.98 | 0.98 |
| 6) 1 mm/pixel; Gaussian(3, 5x5) | 0.99 | 0.98 | 1.00 | 0.98 | 0.98 |
|  |  |  |  |  |  |
| Pixel Size Correction | Fraction of OCCC Values > 0.95 | | | | |
| 1) None | 0.18 | 0.23 | 0.00 | 0.20 | 0.20 |
| 2) 1 mm/pixel | 0.73 | 0.36 | 0.17 | 1.00 | 0.40 |
| 3) Gaussian(sigma=3, mask=5x5) | 0.45 | 0.39 | 0.42 | 0.20 | 0.39 |
| 4) 1 mm/pixel; mean(3x3) | 0.82 | 0.87 | 1.00 | 0.80 | 0.87 |
| 5) 1 mm/pixel; Gaussian(1, 3x3) | 0.91 | 0.94 | 1.00 | 1.00 | 0.93 |
| 6) 1 mm/pixel; Gaussian(3, 5x5) | 1.00 | 0.85 | 1.00 | 0.80 | 0.84 |
|  |  |  |  |  |  |
| Pixel Size Correction | Fraction of OCCC Values > 0.99 | | | | |
| 1) None | 0.09 | 0.14 | 0.00 | 0.00 | 0.12 |
| 2) 1 mm/pixel | 0.27 | 0.17 | 0.00 | 0.00 | 0.16 |
| 3) Gaussian(sigma=3, mask=5x5) | 0.09 | 0.27 | 0.00 | 0.00 | 0.22 |
| 4) 1 mm/pixel; mean(3x3) | 0.09 | 0.55 | 0.50 | 0.20 | 0.50 |
| 5) 1 mm/pixel; Gaussian(1, 3x3) | 0.09 | 0.61 | 1.00 | 0.60 | 0.60 |
| 6) 1 mm/pixel; Gaussian(3, 5x5) | 0.09 | 0.75 | 1.00 | 0.60 | 0.71 |

**S1 Table.** Summary of the OCCC values for 138 radiomics features. This table supports Table 2 in the primary text and shows the results for Gaussian and mean low pass filters rather than Butterworth filters. As indicated in the first column, images were resampled to 1 mm/pixel and were filtered with a mean or Gaussian filter. The masks used to apply the filters to the image pixels were either 3x3 pixels or 5x5 pixels as indicated. The Gaussian filter widths were either 1 or 3 pixels as indicated by the sigma values. GL indicates gray level; NGTDM, neighborhood gray-tone difference matrix; BW, Butterworth; OCCC, overall concordance correlation coefficient.
