# Supplementary material for: Correction: Harmonizing the pixel size in retrospective computed tomography radiomics studies
Source: PLoS One. 2018 Jan 17;13(1):e0191597. doi: 10.1371/journal.pone.0191597 (PMC5771629; doi:10.1371/journal.pone.0191597)
Supplement: S1 Table — This table supports Table 2 in the primary text and shows the results for Gaussian and mean low pass filters rather than Butterworth filters. As indicated in the first column, images were resampled to 1 mm/pixel and were filtered with a mean or Gaussian filter. The masks used to apply the filters to the image pixels were either 3x3 pixels or 5x5 pixels as indicated. The Gaussian filter widths were either 1 or 3 pixels as indicated by the sigma values. GL indicates gray level; NGTDM, neighborhood gray-tone difference matrix; BW, Butterworth; OCCC, overall concordance correlation coefficient. (PDF) [file pone.0191597.s001.pdf]

	Intensity Histogram	GL Co-occurrence	GL Run Length	NGTDM	All Features
	No. of Features				
	11	110	12	5	138
Pixel Size Correction	Mean OCCC Value				
1) None	0.73	0.89	0.83	0.91	0.87
2) 1 mm/pixel	0.95	0.83	0.76	0.96	0.84
3) Gaussian(sigma=3, mask=5x5)	0.75	0.94	0.88	0.85	0.92
4) 1 mm/pixel; mean(3x3)	0.98	0.98	0.99	0.98	0.98
5) 1 mm/pixel; Gaussian(1, 3x3)	0.99	0.98	0.99	0.98	0.98
6) 1 mm/pixel; Gaussian(3, 5x5)	0.99	0.98	1.00	0.98	0.98
Pixel Size Correction	Fraction of OCCC Values > 0.95				
1) None	0.18	0.23	0.00	0.20	0.20
2) 1 mm/pixel	0.73	0.36	0.17	1.00	0.40
3) Gaussian(sigma=3, mask=5x5)	0.45	0.39	0.42	0.20	0.39
4) 1 mm/pixel; mean(3x3)	0.82	0.87	1.00	0.80	0.87
5) 1 mm/pixel; Gaussian(1, 3x3)	0.91	0.94	1.00	1.00	0.93
6) 1 mm/pixel; Gaussian(3, 5x5)	1.00	0.85	1.00	0.80	0.84
Pixel Size Correction	Fraction of OCCC Values > 0.99				
1) None	0.09	0.14	0.00	0.00	0.12
2) 1 mm/pixel	0.27	0.17	0.00	0.00	0.16
3) Gaussian(sigma=3, mask=5x5)	0.09	0.27	0.00	0.00	0.22
4) 1 mm/pixel; mean(3x3)	0.09	0.55	0.50	0.20	0.50
5) 1 mm/pixel; Gaussian(1, 3x3)	0.09	0.61	1.00	0.60	0.60
6) 1 mm/pixel; Gaussian(3, 5x5)	0.09	0.75	1.00	0.60	0.71

Table S1. Summary of the OCCC values for 138 radiomics features. This table supports Table 2 in the primary text and shows the results for Gaussian and mean low pass filters rather than Butterworth filters. As indicated in the first column, images were resampled to 1 mm/pixel and were filtered with a mean or Gaussian filter. The masks used to apply the filters to the image pixels were either 3x3 pixels or 5x5 pixels as indicated. The Gaussian filter widths were either 1 or 3 pixels as indicated by the sigma values. GL indicates gray level; NGTDM, neighborhood gray-tone difference matrix; BW, Butterworth; OCCC, overall concordance correlation coefficient.
