# Supplementary figures and images for: Correction: Harmonizing the pixel size in retrospective computed tomography radiomics studies
Source: PLoS One. 2018 Jan 17;13(1):e0191597. doi: 10.1371/journal.pone.0191597 (PMC5771629; doi:10.1371/journal.pone.0191597)

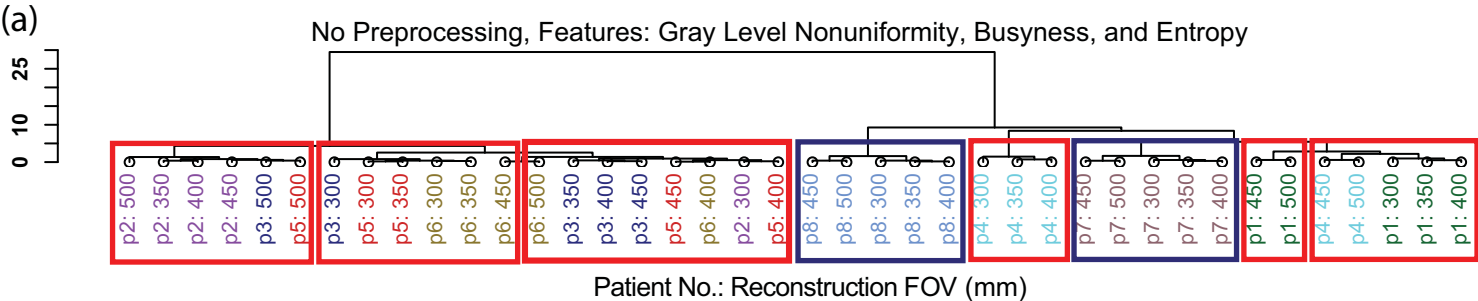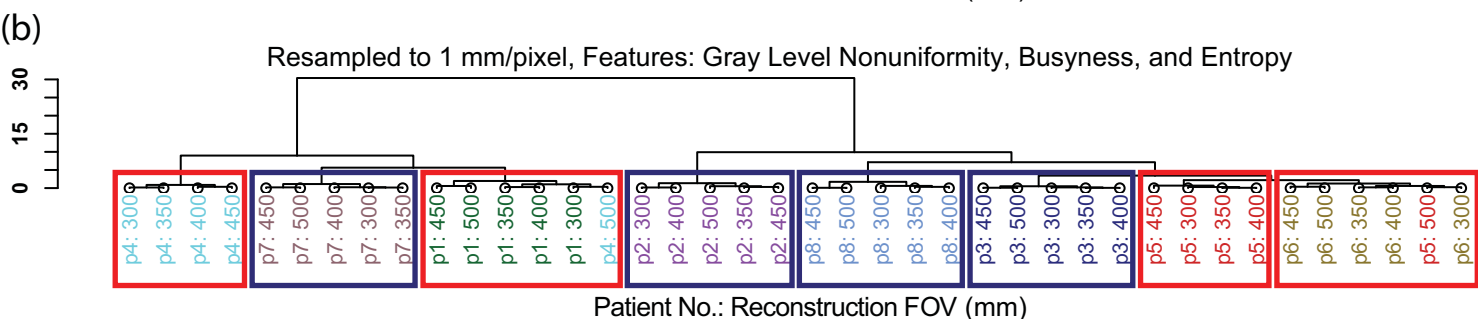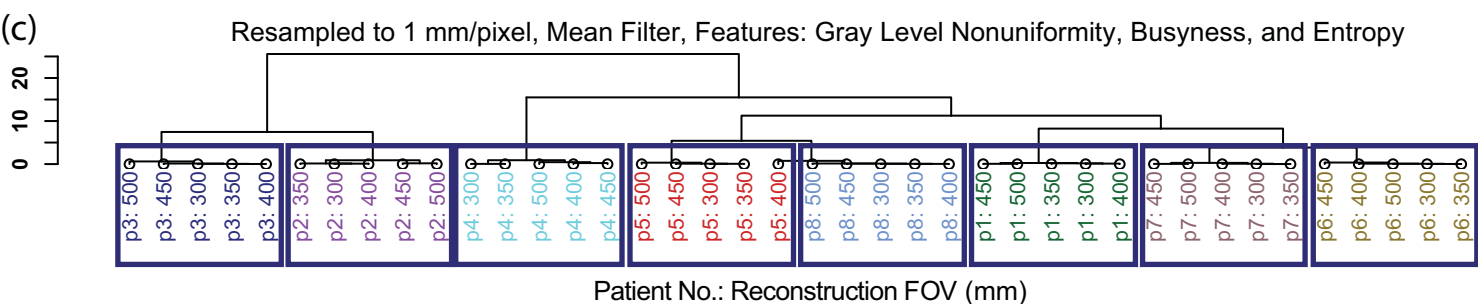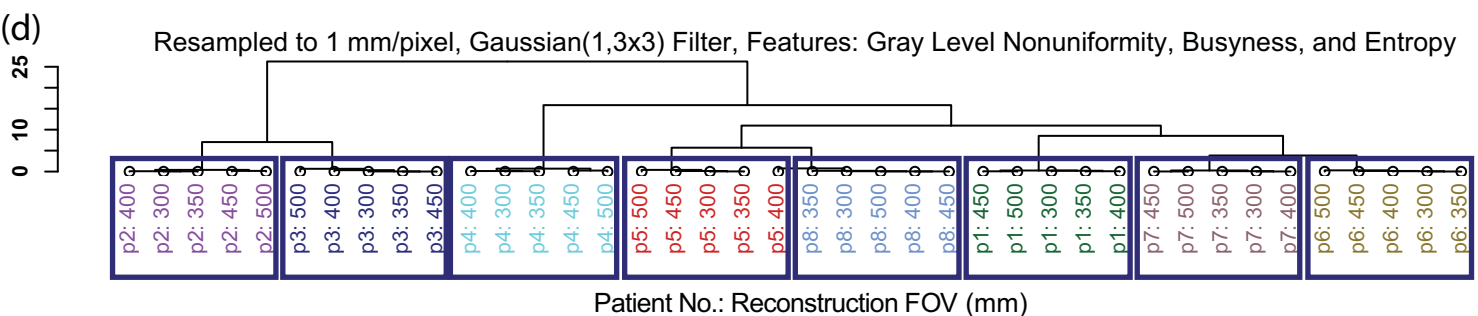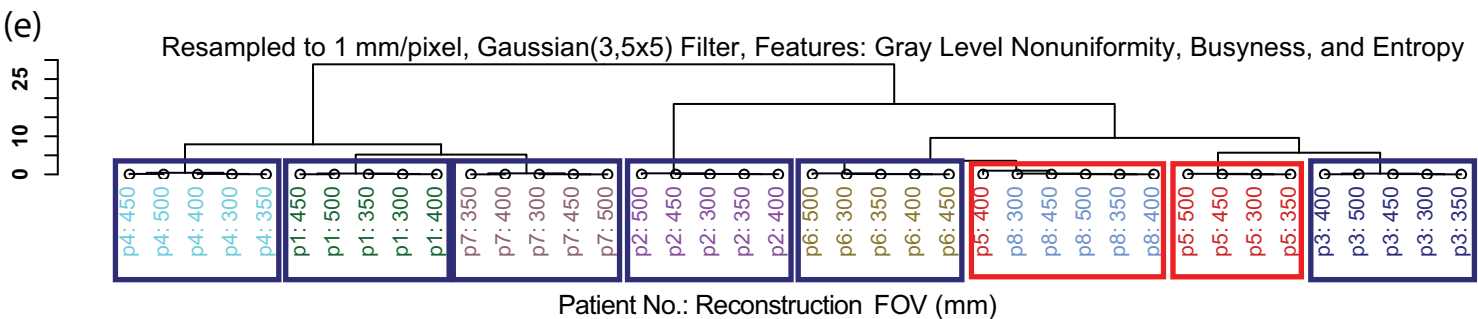

Supplement: S2 Fig — The features were extracted from images that had (a) no preprocessing, (b) resampling to 1 mm/pixel, (c) resampling to 1 mm/pixel and filtering with a 3x3 pixel mean filter, (d) resampling to 1 mm/pixel and filtering with a 3x3 pixel, 1 mm width Gaussian filter, and (e) resampling to 1 mm/pixel and filtering with a 5x5 pixel, 3 mm width Gaussian filter. Boxes indicate incorrect (red) and correct (blue) groupings of the 5 FOV scans for each patient. (PDF) [file pone.0191597.s003.pdf]
