# Supplementary material for: Correction: Harmonizing the pixel size in retrospective computed tomography radiomics studies
Source: PLoS One. 2018 Jan 17;13(1):e0191597. doi: 10.1371/journal.pone.0191597 (PMC5771629; doi:10.1371/journal.pone.0191597)
Supplement: S1 Fig — The features were extracted from images that had (a) no resampling with a Butterworth filter (order 2, frequency cutoff 100), (b) resampling to 1 mm/pixel and filtering with a Butterworth filter (order 2, frequency cutoff 200), (c) resampling to 1 mm/pixel and filtering with a Butterworth filter (order 2, frequency cutoff 125), (d) resampling to 1 mm/pixel and filtering with a Butterworth filter (order 2, frequency cutoff 100), and (e) resampling to 1 mm/pixel and filtering with a Butterworth filter (order 2, frequency cutoff 75). Boxes indicate incorrect (red) and correct (blue) groupings of the 5 FOV scans for each patient. (PDF) [file pone.0191597.s002.pdf]

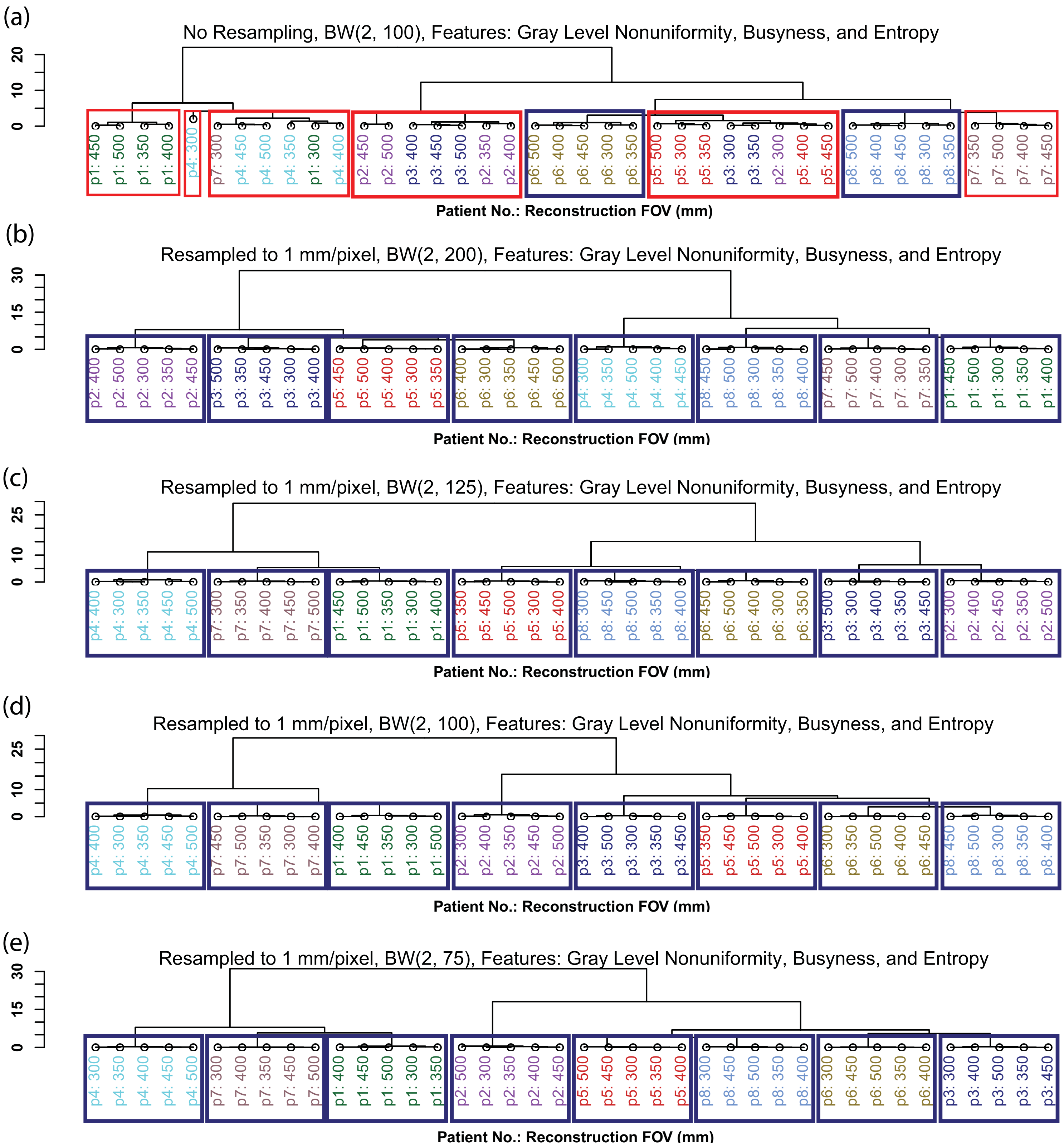
(a)
No Resampling, BW(2, 100), Features: Gray Level Nonuniformity, Busyness, and Entropy
0
10
20
p1: 450
p1: 500
p1: 350
p1: 400
p4: 300
p7: 300
p4: 450
p4: 500
p4: 350
p1: 300
p4: 400
p2: 450
p2: 500
p3: 400
p3: 450
p3: 500
p2: 350
p2: 400
p6: 500
p6: 400
p6: 450
p6: 300
p6: 350
p5: 500
p5: 300
p5: 350
p3: 300
p3: 350
p2: 300
p5: 400
p5: 450
p8: 500
p8: 400
p8: 450
p8: 300
p8: 350
p7: 350
p7: 500
p7: 400
p7: 450
Patient No.: Reconstruction FOV (mm)
(b)
Resampled to 1 mm/pixel, BW(2, 200), Features: Gray Level Nonuniformity, Busyness, and Entropy
0
15
30
p2: 400
p2: 500
p2: 300
p2: 350
p2: 450
p3: 500
p3: 350
p3: 450
p3: 300
p3: 400
p5: 450
p5: 500
p5: 400
p5: 300
p5: 350
p6: 400
p6: 300
p6: 350
p6: 450
p6: 500
p4: 300
p4: 350
p4: 500
p4: 400
p4: 450
p8: 450
p8: 500
p8: 300
p8: 350
p8: 400
p7: 450
p7: 500
p7: 400
p7: 300
p7: 350
p1: 450
p1: 500
p1: 300
p1: 350
p1: 400
Patient No.: Reconstruction FOV (mm)
(c)
Resampled to 1 mm/pixel, BW(2, 125), Features: Gray Level Nonuniformity, Busyness, and Entropy
0
10
25
p4: 400
p4: 300
p4: 350
p4: 450
p4: 500
p7: 300
p7: 350
p7: 400
p7: 450
p7: 500
p1: 450
p1: 500
p1: 350
p1: 300
p1: 400
p5: 350
p5: 450
p5: 500
p5: 300
p5: 400
p8: 300
p8: 450
p8: 500
p8: 350
p8: 400
p6: 450
p6: 500
p6: 400
p6: 300
p6: 350
p3: 500
p3: 300
p3: 400
p3: 350
p3: 450
p2: 300
p2: 400
p2: 450
p2: 350
p2: 500
Patient No.: Reconstruction FOV (mm)
(d)
Resampled to 1 mm/pixel, BW(2, 100), Features: Gray Level Nonuniformity, Busyness, and Entropy
0
10
25
p4: 400
p4: 300
p4: 350
p4: 450
p4: 500
p7: 450
p7: 500
p7: 350
p7: 300
p7: 400
p1: 400
p1: 450
p1: 350
p1: 300
p1: 500
p2: 300
p2: 400
p2: 350
p2: 450
p2: 500
p3: 400
p3: 500
p3: 300
p3: 350
p3: 450
p5: 350
p5: 450
p5: 500
p5: 300
p5: 400
p6: 300
p6: 350
p6: 500
p6: 400
p6: 450
p8: 450
p8: 500
p8: 300
p8: 350
p8: 400
Patient No.: Reconstruction FOV (mm)
(e)
Resampled to 1 mm/pixel, BW(2, 75), Features: Gray Level Nonuniformity, Busyness, and Entropy
0
15
30
p4: 300
p4: 350
p4: 400
p4: 450
p4: 500
p7: 300
p7: 400
p7: 350
p7: 450
p7: 500
p1: 400
p1: 450
p1: 500
p1: 300
p1: 350
p2: 500
p2: 300
p2: 350
p2: 400
p2: 450
p5: 500
p5: 450
p5: 300
p5: 350
p5: 400
p8: 300
p8: 450
p8: 500
p8: 350
p8: 400
p6: 300
p6: 450
p6: 500
p6: 350
p6: 400
p3: 300
p3: 400
p3: 500
p3: 350
p3: 450
Patient No.: Reconstruction FOV (mm)
